# Phenotypic plasticity induced using high ambient temperature during embryogenesis in domesticated zebrafish, *Danio rerio*

**DOI:** 10.1111/rda.13382

**Published:** 2018-12-17

**Authors:** Shahrbanou Hosseini, Bertram Brenig, Jens Tetens, Ahmad Reza Sharifi

**Affiliations:** ^1^ Department of Animal Sciences University of Goettingen Goettingen Germany; ^2^ Center for Integrated Breeding Research University of Goettingen Goettingen Germany; ^3^ Institute of Veterinary Medicine University of Goettingen Goettingen Germany

**Keywords:** embryogenesis, sex determination, sex ratio, survival ability, temperature, zebrafish

## Abstract

Ambient temperature during early stages of life has a substantial effect on physiological processes, eliciting phenotypic plasticity during zebrafish developmental stages. Zebrafish are known to possess a noteworthy ability to modify their phenotype in dependence of environmental factors. However, there is a poor understanding of the effects of temperature during embryogenesis, which influences the biological functions such as survival ability and masculinization in later developmental stages. Since the middle embryonic phase (pharyngula period) is genetically the most conserved stage in embryogenesis, it is very susceptible to embryonic lethality in developmental processes of vertebrates. Here, we tested the effect of transient perturbations (heat shock) during early development (5–24 hr post‐fertilization; hpf) at 35°C compared to control group at 28°C, on survival ability of zebrafish to study the embryonic and post‐embryonic mortality. We studied the variation of heat‐induced masculinization among and across the families in response to high temperature. Furthermore, morphometric traits of adult zebrafish at different developmental time points were measured in order to estimate the temperature × sex interaction effect. We found the highest embryonic mortality around the gastrula and segmentation periods in both experimental groups, with significantly lower survival ability in the temperature‐treated group (73.30% ± 0.58% vs. 70.19% ± 0.57%, respectively). A higher hatching success was observed in the control group (71.08% ± 0.61%) compared to the heat‐induced group (67.95% ± 0.60%). A distinct reduction in survival ability was also observed in both experimental groups during the first two weeks after hatching, followed by a reduced level of changes thereafter. We found sex ratio imbalances across all families, with 25.2% more males under temperature treatment. Our study on growth performance has shown a positive effect of increased temperature on growth plasticity, with a greater impact on female fish in response to high ambient temperature.

## 1 INTRODUCTION

Sex determination (*SD*) is a biological process which determines the sexual fate of an organism and controlled varying widely among different animal species. The underlying factors of this mechanism for sexual decision possess diverse cues such as sex chromosomes, environmental effects, social dynamics and the effects of multiple sex‐associated genes (Webster et al., 2017). Besides the master switch of *SD* that typically resides on well‐differentiated sex chromosomes, different environmental factors can override the genetic influence of *SD* during gonad differentiation in vertebrate species with undifferentiated sex chromosomal pairs (Liew et al., 2012; Liew & Orbán, 2014; Wilson et al., 2014). Zebrafish are widely used as a model animal (Huang, Wang, Zhang, & Wu, 2015; Ribas et al., 2017), and they have no structural differences between the chromosomal sets of the different sexes, and therefore, sex is determined through interaction between the genetic and environmental factors (GXE) (Liew & Orbán, 2014). A recent study in wild‐type and domesticated zebrafish populations, to identify sex‐linked polymorphisms, has reported that chromosome 4 is strongly associated with *SD* in natural populations. Whereas in domesticated populations, no sex‐linked loci have been identified, thus suggesting the *SD* genes are distributed across the genome and sex is determined by a combination of their alleles (Wilson et al., 2014). This type of *SD* is known as polygenic *SD* (PSD) (Liew et al., 2012). Temperature is the most common type of environmental stimulation, inducing masculinization in zebrafish and in many closely related species, in which the sex ratio tends to shift in favour of males (Liew & Orbán, 2014; Ospina‐Alvarez & Piferrer, 2008; Ribas et al., 2017).

During embryogenesis in zebrafish, the primordial germ cells (PGCs) form cell clusters at random locations in the early embryos, before gastrulation, during the first 4 hr of development. The PGCs become motile following specification and migrate towards the somatic gonadal cells, in order to form the primordial gonad, which will then develop into either a testis or an ovary (Liu et al., 2015; Richardson & Lehmann, 2010). This transition completes at the end of the first day of development, whereby the number of PGCs being decisive in determining the sexual fate of the organism (Liu et al., 2015; Raz, 2003; Tzung et al., 2015). In this developmental period, degeneration of PGCs by external environmental stimulations, during cells migration, leads to a decrease in the number of PGCs in the primordial gonad, resulting in masculinization by expression of pro‐male genes and inhibition of ovarian developmental genes (Wang & Orban, 2007; Webster et al., 2017). The influence of high water temperature during larval development in the “juvenile ovary” also leads to oocyte apoptosis and decreased activity of the gonadal aromatase genes during gonad transformation, resulting in masculinization in zebrafish (Liew & Orbán, 2014; Ribas et al., 2017).

Furthermore, the effect of environmental stressor in high and low temperature during embryonic development in terms of survival ability leads to an increase in embryonic mortality, reducing the hatching success, which may be due to the embryo malformations and inability of the emerging larvae to break down the eggshell (Aksakal & Ciltas, 2018). Mid‐embryonic lethality hypothesis predicts an hourglass‐like divergence during animal embryogenesis, in which the embryos in the pharyngula period are highly prone to lethality, compared to the other stages, due to the high conservation of gene expression profiles during this developmental stage (Irie & Kuratani, 2011, 2014). A recent study on zebrafish embryonic mortality highlights that the highest level of embryonic mortality happens during the gastrula period (Uchida, Uesaka, Yamamoto, & Takeda, & H., Irie, N., 2018). Elevated water temperature also influences the larval survival ability after hatching, due to the transition from endogenous to exogenous nutritional sources after depletion of the yolk sac (Wilson, 2012).

As global climate change is predicted to increase water temperatures, which leads to alterations in the ecology and physiology of wild‐type fish species, and increases the risk of extinction in small, inbred populations under natural conditions, due to male‐biased sex ratios and temperature‐induced mortality (Brown et al., 2015; Dorts et al., 2016; Ribas et al., 2017). The main goals of this study were to investigate the effect of exposure of high ambient temperature during embryogenesis on sex ratio imbalances and survival trajectories of zebrafish during embryonic and post‐embryonic development. A further objective of this study was to measure the morphometric traits in different developmental time points of adult zebrafish depending on temperature × sex interaction effect.

## 2 MATERIALS AND METHODS

### 2.1 Animal care and husbandry

The domesticated DDR strain (Von Hertell, Hörstgen‐Schwark, Langholz, & Jung, 1990) of zebrafish was used in this study. All procedures were in strict accordance with the German Animal Welfare Act and national and international recommendations. This study was approved by the University of Goettingen Committee for the care and use of animals (File number E3–17). The fish were kept in recirculation systems of aquaculture facilities, according to the approved institutional guidelines on the use of animals for research purposes (Abozaid, Wessels, & Hörstgen‐Schwark, 2011). The rearing temperature was kept at 28 ± 0.5°C, and fish were subjected to a photoperiod of 12‐hr day/night rhythm in a mixed sex group. The broodstocks were fed twice daily with industrial dry food (Tetramine Junior, Germany) and freshly hatched *Artemia salina *nauplii.

### 2.2 Temperature treatments and data recording

Three consecutive experiments were carried out, during which fertilized eggs from each full‐sib family of zebrafish (69 families) were counted and divided into equal proportions in two experimental groups (control and temperature treatment) from single pair mating. The control group was kept at 28°C, whereas the treatment group was exposed to a high temperature of 35°C from 5 to 24 hpf. This stage of development is the segmentation stage, which occurs between the gastrula period (50% epiboly) and the pharyngula period (Prim‐5) (Abozaid et al., 2011; Kimmel, Ballard, Kimmel, Ullmann, & Schilling, 1995). Water temperature in the incubation system was kept constantly at the required temperature and was controlled three times daily using a digital thermometer (Hobby Biotherm Pro, Gelsdorf, Germany) with an accuracy of ±0.1°C. At the end of treatment period, the temperature of the treated group was gradually reduced to 28°C, the control temperature. The total numbers of eggs were recorded after fertilization and survival ability of incubated eggs during embryonic development in the pre‐treatment (5 hpf) and post‐treatment (24 hpf) stages, as well as the hatchability rate (72 hpf), was recorded by counting and removing the number of dead eggs. Embryonic death was defined as the opaque appearance of eggs, collapse of the egg's structure and unhatched eggs. Feeding started at five days post‐fertilization (dpf), after the yolk sac was absorbed. The larvae were fed three times a day with a commercial food for zebrafish (Tetramine baby, Germany) and *Artemia salina *nauplii. The larvae were maintained in 3‐litre tank (AquaBox® by Aqua Schwarz GmbH, Goettingen, Germany; 23.5 × 13.5 × 13 cm^3^) under a photoperiod of 12‐hr light and 12‐hr darkness with dissolved oxygen around 7 mg/L and pH value of water 7.4 ± 0.2 which was monitored daily (measured using: Multi 3,320, Xylem Analytics, GmbH & Co. KG, WTW, Weilheim, Germany). Other water quality parameters such as ammonia 0.02 mg/L ± 0.01 and nitrite 0.1 mg/L ± 0.01 (measured using Macherey‐Nagel Nanocolor 300D, Dueren, Germany) were controlled periodically to ensure that they were within the appropriate range. The animals of one experiment were examined for their post‐embryonic survival ability, sex ratio and zootechnical parameter (weight, total and standard lengths, survival ability) measurement until sexual maturity. The survival probability of these animals was recorded weekly during the 3 months of age after hatching. The larval survival ability was recorded by counting and removing the number of dead larvae. Zootechnical parameters were measured at 3, 5 and 7 months of age. The phenotypic sex of all individuals in the control and temperature treatment groups was individually determined by the inspection of urogenital papilla. In the case of unclear gonad, the phenotypic sex was determined using microscopic examination. Total and standard lengths of all animals were measured after maturation using a precise digital calliper (Burg‐Wächter PRECISE PS 7,215, Altenhofer, Germany). For the total length, all measurements included the caudal fin, as there was no caudal fin damage and all individuals exhibited normal morphology. The body mass of mature fish was measured using a digital laboratory scales (Scaltec 120 g, Germany).

### 2.3 Statistical analysis

Statistical analysis of the hatchability of survived eggs was carried out by applying a linear logistic model with a binary response variable, which was modelled as a binomial random variable (*y_i_*). The data were then analysed with the GLIMMIX procedure of SAS System 9.3 using the following generalized linear model (Littell, Milliken, Stroup, & Wolfinger, 2006):

$$\log it\left[\frac{\pi_{i}}{1-\pi_{i}}\right]=\eta_{i}=\varphi +\alpha_{i}$$

where *π_i_* is the probability of hatchability at 72hpf of eggs which survived the pre‐treatment stage; *φ* is the overall mean effect; and *α_i_* is the fixed effect of temperature treatment during embryogenesis (*i* = 1: temperature‐treated eggs 35°C and *i* = 2: control group 28°C). Least square means were estimated on the logit scale and then back‐transformed to the original scale (probability) using the inverse link function *π* = exp(*x*)/(1 + (*x*)), where x is the least square means estimate underlying logit scale, applying the LSMEANS statement. Significant differences between least square means were tested using a *t* test procedure by inclusion of the PDIFF option in the LSMEANS statement and adjusted by Tukey–Kramer correction. Standard errors of least square means were calculated as described by Littell et al. (2006). Embryonic survival ability data at different time points of development were analysed using the same statistical model as described above for hatchability data, where *π_i_* is the probability of embryonic survival ability at post‐treatment stage (24 hpf) of eggs that survived in the pre‐treatment stage or probability of survival ability at 72 hpf of eggs that were survived during the post‐treatment stage of development (24 hpf). Statistical analysis of treatment effect on the sex of adult zebrafish was carried out by applying the same statistical model as described above for hatchability data. The dependent variable (*y_i_*) here takes the value 1 for the probability of being male *π_i_* or 0 for the probability of being female 1‐*π_i_* for observation *i*. *α_i_* again represents the fixed effect of temperature treatment during embryogenesis, as described above. The course of larval survival ability during the post‐embryonic time periods until 3 months of age for different treatment groups was illustrated according to the Kaplan–Meier method (survival analysis) by using the LIFETEST procedure of SAS System 9.3 (SAS Institute Inc., USA) and using the following model:

$$\hat{S}\left(t\right)=\prod_{j:t_{j}\leq t}\left[1-\frac{d_{j}}{n_{j}}\right]\text{for}\;t_{1}\leq t\leq t_{k}$$

where *Ŝ*(*t*) is the survivor function and t is the lifetime of animal. For each *j* *t_j_* ≥ *t*, let *t*_1_ <*t*_2_ <… <*t*_k_ representing the different event times. n_j_ is the number of individuals at risk just prior to *t_i_*, and *d_j_* is the number of individuals that die at time *t_j_* (Allison, 2010).

The impact of treatment, sex and different time points of development (age) on zootechnical parameters was analysed using the GLM procedure of SAS with the following model:

$$y_{\mathrm{ijklm}}=\mu +\alpha_{i}+\beta_{i}+\lambda_{k}+\alpha \beta_{\mathrm{ij}}+\alpha \lambda_{\mathrm{ik}}+\beta \lambda_{\mathrm{jk}}+\alpha \beta \lambda_{\mathrm{ijk}}+\delta_{1}+\varepsilon_{\mathrm{ijklm}}$$

where *y_ijklm_* is the observation for a zootechnical parameter, *μ* is the general mean, *α_i_* is the effect of treatment (*i*: 1 = temperature treatment, 2 =  control), *b_j_* is the fixed effect of sex (*j*:1 = male, 2 = female), *l_k_* the fixed effect of time points of development (*k*: 1 = 3 months, 2 = 5 months, 3 = 7 months), *αb_ij_*, *αλ_ik_*, *bλ_jk_* and *αβλ_ijk_* are the fixed effects of interactions, *δ*_l_ is the random effect of tank and *ε_ijklm_* is the random error.

## 3 RESULTS

### 3.1 Embryonic survival ability and hatchability

The descriptive statistics of reproduction traits is presented in Table 1. In this study, 12,222 eggs were derived from 69 full‐sib families in which a total number of 8,177 larvae were hatched after 72 hpf. A total number of 8,718 eggs were alive during the first day (24 hpf) after temperature treatment. This stage of embryonic development is a critical period due to a series of physiological and morphological changes.

**Table 1:** The descriptive statistical phenotypic measurements of the fecundity of the broodstocks and viability of zebrafish eggs derived from a total number of 69 families used in this study during embryonic development until hatching

Variable (time points)	Means of egg numbers	Minimum egg numbers	Maximum egg numbers	Total egg numbers
Fertilized egg number	177.1 ± 11	71	493	12 222
Egg number at 5 hpf	176.3 ± 11	71	491	12 163
Egg number at 24 hpf	126.3 ± 6.4	39	295	8718
Larvae number at 72 hpf	123.9 ± 6.6	37	290	8177

**Figure 1:**
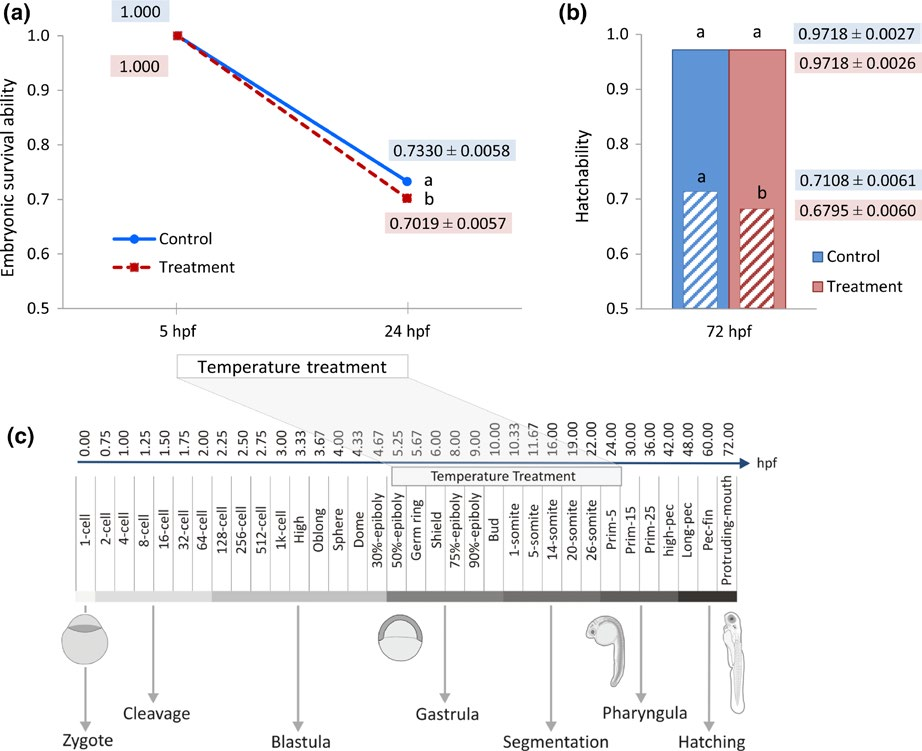
(a) Embryonic survival ability during 24 hr post‐fertilization (hpf) of living eggs in the control and temperature‐treated groups. (b) Hatchability of larvae at 72 hpf in the control and temperature‐treated groups. Pattern‐filled bar chart (box filled with diagonal pattern) illustrates the hatchability of larvae based on the living eggs at the pre‐treatment stage (5hpf), and the coloured bar chart (solid coloured box) shows the hatchability of larvae on the basis of the total number of survived embryos at 24 hpf. ^a–b ^Back‐transformed least square means using generalized linear model within treatment with different superscripts differ significantly (*p* < 0.001). (c) Schematic diagram explains zebrafish embryonic developmental stages sequentially from stages 1 to 35 as described by Kimmel et al. (1995)

The survival ability during the life cycles of zebrafish is categorized into two distinct time periods, embryonic and post‐embryonic stages, regarding developmental morphologies during the zebrafish development. The morphological and developmental processes of zebrafish embryos at different stages as described by Kimmel et al. (1995) and the period of exposure to high ambient temperatures used in this study are illustrated in Figure 1c. In this study, a distinct reduction in survival ability was observed after treatment at 24 hpf in the pharyngula period (Prim‐5, White et al., 2017), compared to the survived eggs at the pre‐treatment (5 hpf) stage in both experimental groups, with a significant (*p* < 0.0002) lower survival ability in temperature‐treated group (73.30% ± 0.58% in control vs. 70.19% ± 0.57% in treated groups). This stage of development is shown to be highly sensitive to fatality independent to elevated water temperature due to high mortality in both treated and non‐treated groups (Figure 1a). The hatchability rate was recorded at 72 hpf, in which the larvae were swimming free out of their chorion. A significant (*p* < 0.0003) higher hatching success was observed in the control group (71.08% ± 0.61%) compared to temperature treatment group (67.95% ± 0.60%) based on the living eggs at the pre‐treatment stage (5 hpf). This result is mainly a consequence of the number of pre‐stage surviving embryos at 24 hpf. However, no significant differences in hatching rates were found between the two experimental groups on the basis of the total number of survived embryos at 24 hpf (97.18% ± 0.27% in control vs. 97.18% ± 0.26% in treated groups), meaning that the temperature has no effect on hatchability after treatment period (Figure 1b).

### 3.2 Time‐course survival of post‐embryonic development

**Figure 2:**
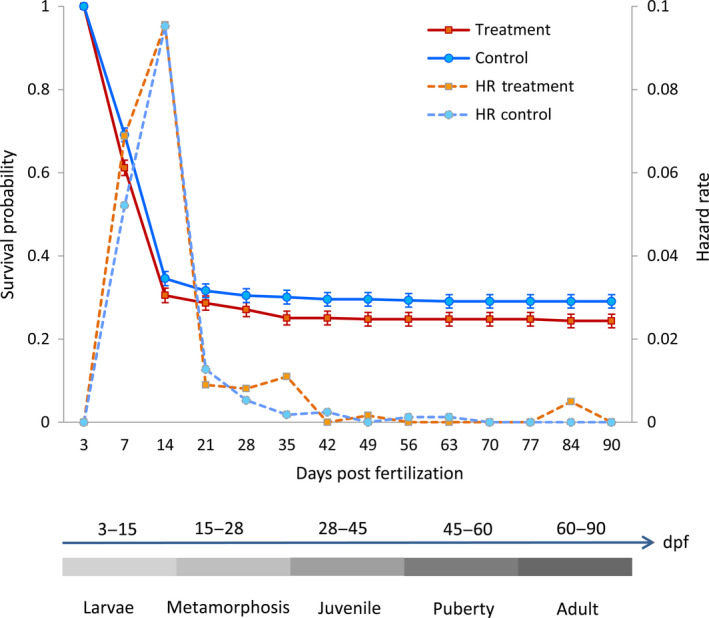
Kaplan–Meier survival curves and hazard rate illustrate the survival ability of hatched larvae during the post‐embryonic development from 3 days post‐fertilization (dpf) until sexual maturity at 90 dpf. The survival curves of different treatment differ significantly using Wilcoxon test (*p* < 0.001). Post‐embryonic developmental stages of zebrafish until adulthood are represented in schematic diagram as defined by Ribas and Piferrer (2014)

The effect of elevated water temperature on the survival rate of zebrafish during a series of developmental stages was continuously investigated up to the adult stage (Figure 2). The survival probability, using Kaplan–Meier (KM) survival curves, shows highly significant differences (*p* < 0.001) between control and temperature‐treated groups. The result presented here reveals the obvious decrease in survival ability in both temperature treatment and control groups during the first 2 weeks post‐hatching, meaning increased risk of mortality (Hazard rate). Interestingly, after this period, the KM survival curves are illustrated steady lower changes as the number of day increases until 90 dpf in both experimental groups. Despite a constant survival rate in both treated and non‐treated groups, we observed a higher mortality in the temperature‐treated group, which caused by past survival ability at 14 dpf and lasts until 3 months of age.

### 3.3 Family‐specific sex ratio in different temperature treatment

**Figure 3:**
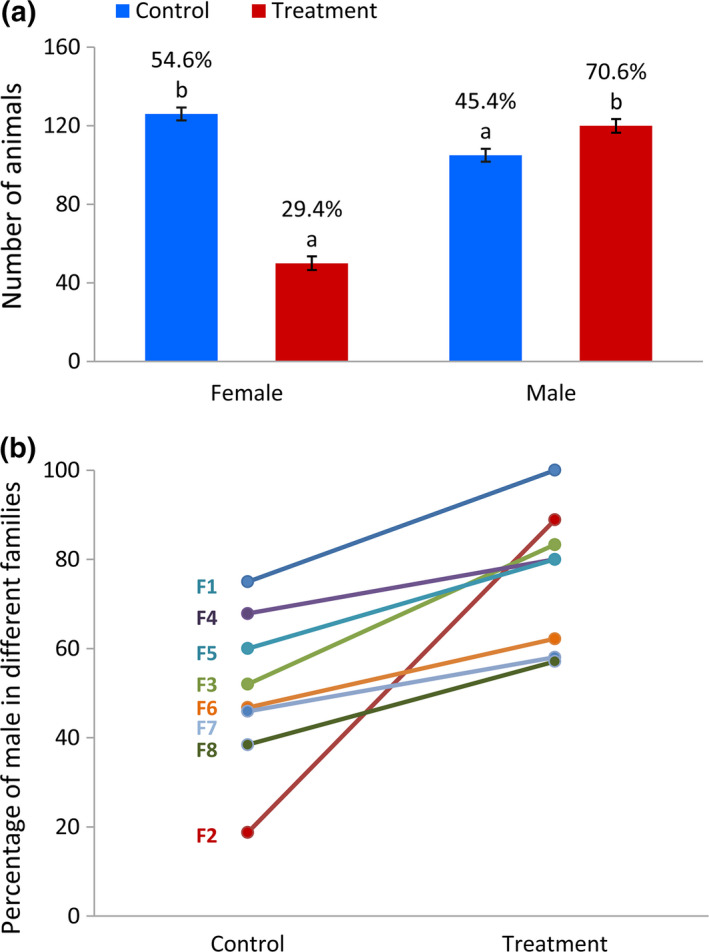
(a) Sex ratio (back‐transformed least square means in % using generalized linear model) in control and temperature‐treated groups across all families. (b) Sex ratio in different families (F) in control and temperature‐treated groups (back‐transformed least square means in % using generalized linear model). ^a–b^Means within treatment with different superscripts differ significantly (*p* < 0.0001)

The sex ratio of adult fish across all families, and family‐specific, is shown in Figure 3. The effect of elevated water temperature during embryogenesis indicated a significantly (*p* < 0.0001) higher proportion of males in the temperature‐exposed animals compared to the control group (70.6% ± 3.49% vs. 45.4% ± 3.27%, Figure 3a). The results also show a wide range of interfamily sex ratio variation in response to temperature with interplaying between genetic and environmental factors (G × E), implying a PSD‐induced masculinization during gonad differentiation in domesticated zebrafish (Figure 3b). A higher male ratio in all families was observed in the temperature‐treated groups compared with their corresponding control groups. The effect of increased water temperature is presented in female‐biased families (more than 50% female in the control group) as well as in male‐biased families (more than 50% male in the control group) in family‐specific sex ratio result.

### 3.4 Morphometric traits at different time points of zebrafish development

Table 2 summarizes the least square means and the significance of explanatory variables on body mass, and total and standard lengths of adult zebrafish. For all three variables, a significant effect of the main factors, sex and treatment, as well as an interaction between these main factors, was determined. Comparison of the weight of males in treatment and control groups revealed that there is no influence of temperature on the development of body weight (0.3400 ± 0.011 vs. 0.3427 ± 0.011). However, the temperature treatment caused a significant increase (*p* < 0.0001) in female body weight, when compared with males, and also compared with the weight of non‐treated female animals (0.5065 ± 0.013 females in treated vs. 0.4156 ± 0.011 females in control). A considerably higher body mass was observed in females compared to males (0.4611 ± 0.010 vs. 0.3413 ± 0.009), resulted in significant effect (*p* < 0.0001) of the main factor sex and treatment. Exposing the embryos to high water temperature, regardless of sex, leads to increased body weight (0.4232 ± 0.010 vs. 0.3791 ± 0.009). The same is true if one looks at means derived from three‐way interactions (sex × treatment × age).

**Table 2:** Least square means, standard error (±*SE*) and level of significance for body mass (g), and total and standard lengths (mm) in adult zebrafish for the effect of temperature, sex, age and their interactions

Effect	Traits		
	Body mass (g)	Total length (mm)	Standard length (mm)
Sex			
Female (F)	0.4611 ± 0.010^a^	33.98 ± 0.221^a^	27.74 ± 0.203^a^
Male (M)	0.3413 ± 0.009^b^	33.15 ± 0.191^b^	27.08 ± 0.173^b^
Temperature treatment			
Control (C)	0.3791 ± 0.009^a^	33.15 ± 0.191^a^	27.07 ± 0.173^a^
High (H)	0.4232 ± 0.010^b^	33.98 ± 0.222^b^	27.76 ± 0.203^b^
Age			
3 months (3 M)	0.3450 ± 0.012^a^	31.92 ± 0.281^a^	26.31 ± 0.254^a^
5 months (5 M)	0.4030 ± 0.012^b^	33.78 ± 0.290^b^	27.46 ± 0.262^b^
7 months (7 M)	0.4555 ± 0.007^c^	34.99 ± 0.172^c^	28.47 ± 0.155^c^
Sex × Treatment			
F × C	0.4156 ± 0.011^a^	33.26 ± 0.276^a^	27.12 ± 0.250^a^
M × C	0.3427 ± 0.011^b^	33.03 ± 0.263^a^	27.02 ± 0.238^a^
F × H	0.5065 ± 0.013^c^	34.69 ± 0.345^b^	28.37 ± 0.312^b^
M × H	0.3400 ± 0.011^b^	33.28 ± 0.278^a^	27.15 ± 0.252^a^
Treatment × Age			
C × 3 M	0.3330 ± 0.015	31.72 ± 0.375	26.18 ± 0.339
C × 5 M	0.3809 ± 0.015	33.27 ± 0.386	27.04 ± 0.349
C × 7 M	0.4235 ± 0.008	34.44 ± 0.194	28.98 ± 0.176
T × 3 M	0.3571 ± 0.016	32.11 ± 0.419	26.44 ± 0.379
T × 5 M	0.4251 ± 0.016	34.30 ± 0.430	27.87 ± 0.391
T × 7 M	0.4875 ± 0.011	35.54 ± 0.284	28.96 ± 0.256
Sex × Age			
F × 3 M	0.3958 ± 0.015^bc^	32.39 ± 0.402	26.74 ± 0.363
F × 5 M	0.4491 ± 0.017^b^	33.99 ± 0.447	27.54 ± 0.404
F × 7 M	0.5382 ± 0.011^a^	35.55 ± 0.282	28.95 ± 0.254
M × 3 M	0.2942 ± 0.015^d^	31.44 ± 0.393	25.88 ± 0.355
M × 5 M	0.3569 ± 0.014^c^	33.58 ± 0.370	27.38 ± 0.334
M × 7 M	0.3728 ± 0.008^c^	34.44 ± 0.197	28.00 ± 0.178
Sex × Treatment × Age			
F × C × 3 M	0.3662 ± 0.020	31.73 ± 0.544	26.16 ± 0.491
F × C × 5 M	0.4025 ± 0.021	33.22 ± 0.575	26.87 ± 0.500
F × C × 7 M	0.4780 ± 0.010	34.83 ± 0.250	28.31 ± 0.226
F × T × 3 M	0.4255 ± 0.022	33.06 ± 0.593	27.32 ± 0.536
F × T × 5 M	0.4958 ± 0.025	34.76 ± 0.684	28.21 ± 0.618
F × T × 7 M	0.5984 ± 0.018	36.26 ± 0.505	29.59 ± 0.457
M × C × 3 M	0.2997 ± 0.020	31.72 ± 0.517	26.19 ± 0.467
M × C × 5 M	0.3593 ± 0.019	33.32 ± 0.517	27.21 ± 0.467
M × C × 7 M	0.3690 ± 0.012	34.05 ± 0.298	27.65 ± 0.270
M × T × 3 M	0.2888 ± 0.022	31.17 ± 0.592	25.57 ± 0.536
M × T × 5 M	0.3544 ± 0.020	33.84 ± 0.530	27.54 ± 0.479
M × T × 7 M	0.3766 ± 0.010	34.82 ± 0.258	28.33 ± 0.233

ANOVA, *P*‐value (*F*)	Weight	Total length	Standard length
Sex	≤0.0001	≤0.0053	≤0.0131
Temperature treatment	≤0.0001	≤0.0044	≤0.0093
Age	≤0.0001	≤0.0001	≤0.0001
Sex × Treatment	≤0.0001	≤0.0441	≤0.0339
Treatment × Age	≤0.2305	≤0.5511	≤0.4887
Sex × Age	≤0.0018	≤0.5804	≤0.4228
Sex × Treatment × Age	≤0.6699	≤0.6549	≤0.6084

Note

Means within a factor with different superscripts letters differ (*p* < 0.05).

A similar sex x treatment interaction was found for total and standard lengths, although no differences were observed between female and male animals in the control group. The highest total and standard lengths were observed for treated females, when compared with animals of other groups. A significant increase is shown in the total and standard lengths of female animals compared to male animals (33.98 ± 0.221 vs. 33.15 ± 0.191, *p* < 0.0053; 27.74 ± 0.203 vs. 27.08 ± 0.173, *p* < 0.0131) and treatment compared to control (33.98 ± 0.222 vs. 33.15 ± 0.191, *p* < 0.0044; 27.76 ± 0.203 vs. 27.07 ± 0.173, *p* < 0.0093). At all three age points, the female animals in the treatment group show higher body weight, total and standard lengths compared with animals of other groups, resulting in non‐significant three‐way interactions. This shows that the effect of temperature treatment on weight development and length growth in females begins, and develops more rapidly over the experimental period, in comparison with males which begin at a lower level and do not increase as rapidly as females. This trend can also be observed when looking at the sex x age interaction, in which the females show a higher weight and length development than the male animals, when the animals become older.

## 4 DISCUSSION

### 4.1 Embryonic and post‐embryonic survival ability

In this study, the effect of high ambient temperature during embryogenesis on reproductive, survival ability, sex ratio imbalances and morphometric traits during zebrafish developmental stages were investigated. The fecundity of zebrafish is relatively low, compared to other fish species, with a maximum of 500 eggs per clutch due to their small size. A larger clutch size of laboratory zebrafish females was observed than wild population (Ribas & Piferrer, 2014), which is in agreement with the fecundity ability of laboratory strain used in this study.

The first stage of development (1.5 hpf) is the fourth phase of cleavage and produces a set of 4 × 4 arrays of blastomeres cells (16 cells) in the zygote. However, in the second developmental period (5 hpf), epiboly displays blastoderm margin between the animal and vegetal pole. In the pharyngula period, a variety of morphogenetic movements occur and the primary organs become visible, in particular, the tail bud becomes increasingly distinguished and the first body movements appear (Kimmel et al., 1995). In evolutionary developmental biology, the hourglass model of embryonic evolution in vertebrates predicted the mid‐embryonic phase, namely the phylotypic period, to be the most conserved organogenesis stage compared to earliest and latest stages of embryo development. Transcriptome profile during mid‐embryonic phase (pharyngula period) in vertebrates is highly conserved, which provides a gene source for the development of the basic body plan (Irie & Kuratani, 2011, 2014). Therefore, it was hypothesized that increased embryonic mortality would potentially occur during this conserved developmental period (Irie & Kuratani, 2011; Uchida et al., 2018). However, in the study completed by Uchida et al. (2018), the embryonic mortality was higher in earlier stages of development around the gastrula and early segmentation periods, which is in agreement with the results of our study. In the same study, increased embryonic mortality was observed during the gastrula stage with high‐temperature treatment as a thermal perturbation, emphasizing the sensitivity of these stages to environmental factors (Uchida et al., 2018). In a comparable study, carried out on eggs deriving from the mating of mitotic gynogenic male with normal female, a low hatching rate at 72 hpf was reported in temperature‐induced zebrafish compared to control group (16% vs. 27%) that were treated by high water temperature (35°C) at 5–24 hpf during embryogenesis. However, there is no information provided from the authors regarding embryonic survival ability during previous stages of development, before the hatching process started (Abozaid et al., 2011). A pronounced adverse effect of increased water temperature as a perturbation factor in the embryogenesis process leading to increased mortality before 24 hpf was also observed in the study of Aksakal and Ciltas (2018).

Cooling exposure at 50% epiboly stage for 6 and 18 hr in zebrafish embryos also resulted in a considerably reduced embryonic survival ability (control: 67.50% vs. 18 hr: 39.80% and 6 hr: 18.75%) during hatching process (Paes & Nakaghi, 2017). From the embryos surviving the gastrulation stage, a further reduction in hatchability in the post‐gastrulation stage, under the influence of high water temperature, may be due to the inhibition of mitosis, the inhibition of the hatching enzyme activity, suppression of embryogenesis or an inability of the emerging larvae to break down the eggshell along with malformations such as pericardial and yolk‐sac oedema, tail deformity and spinal curvature (Aksakal & Ciltas, 2018).

The early larvae stage in zebrafish lasts up to two weeks following hatching (~15 dpf; Ribas & Piferrer, 2014), involving developmental stages such as yolk‐sac larvae (~4 dpf) and swim‐up larvae (~9 dpf) characterized by physiological and morphological changes (Bagatto, Pelster, & Burggren, 2001). The swim bladder inflates during 4–5 dpf and they can start short burst swimming, whereas the digestive tract opens and the digestive enzymes are secreted during the 5–6 dpf, suggesting the larvae can feed exogenously from endogenous feeding (Wilson, 2012). Larvae are more vulnerable during these developmental stages due to the depletion of yolk sac, inflation of the swimming bladder and swimming performance influencing the food capture (Sfakianakis, Leris, & Kentouri, 2011; Wilson, 2012). Furthermore, the early life temperature treatment could have an effect on fish muscle ontogeny or body shape (Sfakianakis et al., 2011), which impacts the swimming ability in water current direction. The larval stage (takes up to 2 weeks) involves changes in a wide range of traits such as development of scales, pigmentation patterns and gut tube drops more ventrally (Ribas & Piferrer, 2014). This wide range of morphological and physiological changes might be the cause of increased mortality in this stage of development, which is observed in our study, in particular in the case of treated animals. In the later stage of development, free‐swimming larvae (~21 dpf: mid‐stage larvae, Bagatto et al., 2001; Wilson, 2012) start metamorphosis between 15 and 28 dpf. In our study, this developmental process has no particular effect on larval survival ability.

### 4.2 Sex ratio response to environmental temperature

In this study, the effect of elevated temperature during early zebrafish life resulted in sex ratio imbalances with a higher proportion of males in heat‐exposure animals. A similar study of elevated water temperature on the golden strain of zebrafish during different embryonic developmental stages (5–10, 5–24 and 5–48 hpf) at 35°C in half‐sib families derived from mating of mitotic gynogenic males with normal females resulted in a higher proportion of males by 25% in 5–24 hpf (22% in control vs. 47.54% in treatment), although a higher proportion of females in the progeny generation was expected (Abozaid et al., 2011). The results reported in this study, in terms of masculinization, are in agreement with our study.

Heat‐induced masculinization due to temperature treatment at the larval stage, between 18 and 32 dpf in the AB strain of domesticated zebrafish, is also showed that the sex ratio varies considerably among different families (Ribas et al., 2017). The result of the study of Ribas et al. (2017) revealed that some heat‐treated animals altered their sex from genotypic females to phenotypic males, these individuals being known as “neomales,” and the remaining genotypic females can be considered as heat‐resistant individuals or “superfemales.” Neomales possess testis and have similar gene expression profiles to normal males, but the underlying genetic mechanism for *SD* of these animals and the heat‐resistant females is so far unknown (Ribas et al., 2017). Therefore, the result of our study in PSD confirms previous observations of environmental dependency of sexual plasticity in domesticated zebrafish. Phenotypic masculinization is also reported in many other fish species with similar *SD* plasticity, such as European sea bass, *Dicentrarchus labrax* (Díaz & Piferrer, 2015), medaka, *Oryzias latipes* (Selim, Shinomiya, Otake, Hamaguchi, & Sakaizumi, 2009), and Nile tilapia, *Oreochromis niloticus* (Rougeot, Prignon, Kengne, & Mélard, 2008).

PGCs play an important role in gonad differentiation and sexual dimorphism (Liu et al., 2015; Tzung et al., 2015). Elevated water temperature is the most important environmental parameter, may induce degeneration of PGCs, influencing the genetic mechanism of *SD*, resulting in masculinization (Lee et al., 2009; Selim et al., 2009). The genetic regulation of *SD* in juvenile ovaries during gonad development and sexual polymorphism is controlled by the major pro‐male (e.g., *dmrt1, amh and cy11c1*) and pro‐female genes (e.g., *cyp19a1a, foxl2 and vtg2*). In the masculinization process, expression of *dmrt1*, a key regulator of *SD* gene in males, activates *amh*, which inhibits the ovarian aromatase gene expression and oocyte apoptosis, resulting in ovary‐to‐testis transformation (Lee et al., 2017; Wang & Orban, 2007; Webster et al., 2017). In this process, upregulation of the most important pro‐male pathway (Tp53‐activated apoptosis) and downregulation of pro‐female pathways (NF‐κB and canonical Wnt) caused a shift of the sex ratio towards an increased proportion of males (Liew & Orbán, 2014).

### 4.3 Morphometric traits

High and low temperatures (32°C and 22°C compared to the control at 27°C) during embryonic development in zebrafish have an influence on the acclimation response, which has an influence on energy metabolism and swimming performance in later life stages, which is controlled by expression of several metabolic genes (Scott & Johnston, 2012) in swimming muscle (Schnurr, Yin, & Scott, 2014). In the study of Schnurr et al. (2014), temperature treatment during early life in zebrafish resulted in a reduction in body mass and standard length until 12 weeks of age. However, in the high‐temperature group, a significant growth rate was observed beyond this age and the body length was higher than the control group (Schnurr et al., 2014), which is in agreement with our study. The influence of different temperature ranges during embryonic development on post‐hatching growth rate in other fish species has also been reported (Macqueen et al., 2008). The effect of different temperature (low temperature: 18°C and high temperature: 22°C) during embryogenesis on muscle growth rate and body mass in gilthead sea bream showed that early temperature treatment has an influence on the expression patterns of a subset of muscle developmental genes (*Hsp90a, UNC45, MyoD *and *IGF1*) and their expression is modified by different temperature regimes (Serrana et al., 2012). Interestingly, a positive effect of elevated water temperature on growth rate was observed during post‐embryonic development when the animals were reared at three different temperatures: 24°C, 28.5°C and 33°C. Animals reared at 24°C grew slower than those reared at 33°C (Parichy, Elizondo, Mills, Gordon, & Engeszer, 2009). Our study also demonstrated a positive effect of increased temperature on growth performance, with a higher influence on females in response to high ambient temperature.

## 5 CONCLUSION

Exposure to high water temperature during embryonic development in domesticated zebrafish demonstrated the reduction in survival ability in the first day after fertilization and the first two weeks after hatching, which shows the most sensitive life stages to thermal changes in the environment. Furthermore, the effect of elevated water temperature during embryogenesis resulted in sex ratio imbalances with more males under high‐temperature condition. Our study on growth performance in different developmental life cycles in heat‐induced domesticated zebrafish during adult stages after sexual maturity has shown the growth plasticity in response to high ambient temperature for different sexes. A positive effect of increased temperature during embryogenesis on growth with a greater impact in female fish is observed in this study.

## CONFLICT OF INTEREST

The authors declare that no conflicting personal or financial interests.

## AUTHOR CONTRIBUTIONS

S.H. contributed to the conception and design of the study, carried out the experiments, interpreted the results and wrote the manuscript. A.R.S. designed the study and conception. S.H. and A.R.S. performed computational data analysis and interpreted the data. B.B., J.T. and A.R.S. edited and corrected the manuscript. All authors read and commented on the manuscript and approved the final version.
